# Supplementary material for: Cascade Testing for Fragile X Syndrome in a Rural Setting in Cameroon (Sub-Saharan Africa)
Source: Genes (Basel). 2020 Jan 28;11(2):136. doi: 10.3390/genes11020136 (PMC7074341; doi:10.3390/genes11020136)
Supplement: Supplementary file 1 [file genes-11-00136-s001.pdf]

Table S1. Summary classification of Intellectual Disability using four different scales.

Severity Category	Approximate Percent Distribution of Cases by Severity	DSM-IV Criteria (severity levels based only on IQ categories)	DSM-5 Criteria (severity classified based on daily skills)	AAIDD Criteria (severity classified based on intensity of support needed)	SSI Listings Criteria (The SSI listings do not specify severity levels but indicate different standards for meeting or equaling listing level severity.)
**Mild**	85%	Approximate IQ range 50–69	Can live independently with minimum levels of support.	Intermittent support needed during transitions or periods of uncertainty.	IQ of 60 through 70 *and* a physical or other mental impairment imposing an additional and significant limitation of function
**Moderate**	10%	Approximate IQ range 36–49	Independent living may be achieved with moderate levels of support, such as those available in group homes.	Limited support needed in daily situations.	A valid verbal, performance, or full-scale IQ of 59 or less
**Severe**	3.5%	Approximate IQ range 20–35	Requires daily assistance with self-care activities and safety supervision.	Extensive support needed for daily activities.	A valid verbal, performance, or full-scale IQ of 59 or less
**Profound**	1.5%	IQ <20	Requires 24-hour care.	Pervasive support needed for every aspect of daily routines.	A valid verbal, performance, or full-scale IQ of 59 or less
